# MRI-based deep learning model for early TACE response prediction in HCC: multicenter validation with biological insights

**DOI:** 10.1186/s12885-025-15273-8

**Published:** 2025-11-24

**Authors:** Mingzhen Chen, Zhongwei Zhao, Lingling Zhou, Chunli Kong, Xinyu Guo, Weiyue Chen, Guihan Lin, Xia Li, Liyun Zheng, Shuiwei Xia, Chenying Lu, Xiaoxi Fan, Minjiang Chen, Zhiyi Peng, Jiansong Ji

**Affiliations:** 1 Zhejiang Key Laboratory of Imaging and Interventional Medicine, Zhejiang Engineering Research Center of Interventional Medicine Engineering and Biotechnology, School of Medicine, Lishui Hospital, Zhejiang University, 289 Kuocang Road, Lishui, 323000 China; 2 Department of Radiology, Lishui Central Hospital, the Fifth Affiliated Hospital of Wenzhou Medical University, 289 Kuocang Road, Lishui, 323000 China; 3 Department of Radiology, The Fifth Affiliated Hospital of Sun Yat-Sen University, Zhuhai, Guangdong 519000 China; 4 Imaging Intervention Division, Affiliated Hangzhou First People’s Hospital, School of Medicine, Westlake University, Hangzhou, 310006 China; 5 Division of Hepatobiliary and Pancreatic Surgery, Hepatobiliary and Pancreatic Interventional Treatment Center, The First Affiliated Hospital, Zhejiang University School of Medicine, Hangzhou, 310003 China

**Keywords:** Hepatocellular carcinoma, Transarterial chemoembolization, MRI, Deep learning, Treatment response

## Abstract

**Background:**

Transarterial chemoembolization (TACE) remains a cornerstone treatment for hepatocellular carcinoma (HCC), yet heterogeneous treatment response poses significant clinical challenges. This multicenter study aimed to develop and validate a deep learning model that leverages pretreatment MRI to predict objective response to initial TACE, while exploring imaging-biological correlations.

**Methods:**

We utilized retrospective data from 3 institutions, which included HCC patients who underwent TACE. A deep learning algorithm (hereinafter, DLTR) was developed for predicting TACE response by comparing various deep learning algorithms. A multilayer perceptron was then employed to integrate potential clinical factors into the model (hereinafter, DLTR_MLP_) classifier. Performance was evaluated by the area under the receiver operating characteristic curve (AUC) in internal and external cohorts. Survival differences were assessed using log-rank test in two external test sets. RNA-sequencing data from the Cancer Image Archive (TCIA) were used to link imaging signatures to biological pathways.

**Results:**

DLTR_MLP_ achieved higher AUC than DLTR and clinical models in predicting TACE efficacy in two external test cohorts (AUC: 0.8 vs. 0.649, 0.648; 0.818 vs. 0.629, 0.659) and effectively stratified patients by progression-free survival (*P* = 0.035). Deep learning features correlated with 149 genes (*P* < 0.05), which were notably enriched in angiogenesis, EMT, hypoxia, and TGF-β Signalling pathways.

**Conclusion:**

The DLTR_MLP_ model, combining MRI-based deep learning and clinical variables, robustly predicts TACE response and reveals imaging signatures linked to tumour proliferation biology. Its potential integration into MRI workflows could help optimize treatment decision-making for HCC.

**Supplementary Information:**

The online version contains supplementary material available at 10.1186/s12885-025-15273-8.

## Introduction

Transarterial chemoembolization (TACE) is currently regarded as one of the most generally used treatments for intermediate and advanced hepatocellular carcinoma (HCC) as per the Barcelona Clinic Liver Cancer (BCLC) staging system [1]. However, the efficacy of TACE shows significant inter-individual variability. According to a large systematic review, the objective response rate for TACE was 52.5%. The overall survival rate was 70.3% at 1 year, 51.8% at 2 years, 40.4% at 3 years, and 32.4% at 5 years [2]. These statistics indicate that nearly 30% of patients have a poor prognosis, while 32.4% of patients are still alive after 5 years. Despite careful patient selection, the therapeutic effect of TACE varies significantly. Effective TACE treatment can prevent tumour progression and hopefully achieve tumour downgrading, providing opportunities for surgical resection [3, 4]. However, TACE-insensitive patients may experience a decrease in liver function reserve or even liver dysfunction after multiple TACE treatments, resulting in a decline in the quality of life of patients and missed the opportunity to receive targeted kinase inhibitors treatments. This highlights the need for more precise predictive strategies to select patients who may benefit from TACE procedure.

Several clinical parameters, such as the absence of portal vein tumour thrombosis, high serum bilirubin, and lower alpha-fetoprotein level, are associated with a higher likelihood of TACE response [5]. Researchers have found that certain imaging semantic features, such as lesion size, margin, and enhancement pattern, may be associated with TACE efficacy [6]. At the molecular level, the heterogeneity of tumour biological behaviour is important factor affecting TACE response. Studies have shown that vascular endothelial growth factor (VEGF) and heparin-binding epidermal growth factor (HB-EGF) are sensitive markers of invasion, recurrence and prognosis of HCC [7, 8]. Studies focused on TACE have also tried to analyse these factors, but failed to provide precise results [9]. In addition, histopathological results, such as microvascular invasion and macrotrabecular-massive subtype, are also associated with proliferation, metastasis, and poor prognosis of HCC [10, 11]. However, due to the inaccessible of surgical specimens during TACE, and the relationship between tumour biological information and TACE efficacy is still unclear.

Convolutional neural networks (CNNs) are powerful deep learning algorithms that can recognize patterns in medical images through convolution operations. Emerging evidence demonstrated the feasibility of using CNNs to perform various tasks from medical images, such as tumour diagnosis, treatment planning, and prognostic evaluation [12–14]. Their application to TACE efficacy prediction remains underexplored-a gap with direct clinical implications. To address these challenges, ​​this study introduces a novel deep learning tumour response (DLTR) model that integrates multiparametric MRI features with key clinical variables.​​ Unlike prior studies that have relied on either clinical parameters or conventional imaging semantic features in isolation, our DLTR model leverages a CNN for MRI analysis coupled with a multilayer perceptron (MLP) for clinical data, ​​thereby capturing complex nonlinear relationships to improve prediction accuracy [15].​​ Furthermore, ​​multicentre validation was conducted to strengthen the generalizability and clinical translation potential of our findings. Finally, we explore transcriptomic signatures in a patient subgroup to uncover the biological mechanisms underlying MRI-based predictions of TACE resistance​​, moving beyond pure prediction towards mechanistic insight. By enabling the early identification of optimal TACE candidates, this integrated approach aims to refine therapeutic decision-making, conserve liver function in potential non-responders, and ultimately improve survival trajectories in HCC management.

## Methods and materials

This retrospective three-institution study was approved by the Institutional Review Boards of Lishui Central Hospital (institution 1), The First Affiliated Hospital, Zhejiang University School of Medicine (institution 2), and the Hangzhou First People’s Hospital (institution 3). Due to the retrospective nature of the study, the requirement for patient-informed consent was waived. The study was carried out in agreement with the TRIPOD Checklist (www.tripod-statement.org). The Chinese Clinical Trial Registry institution approved and registered this study (ChiCTR2400079714) on 10 January 2024 [16].

### Study design

**Fig. 1:**
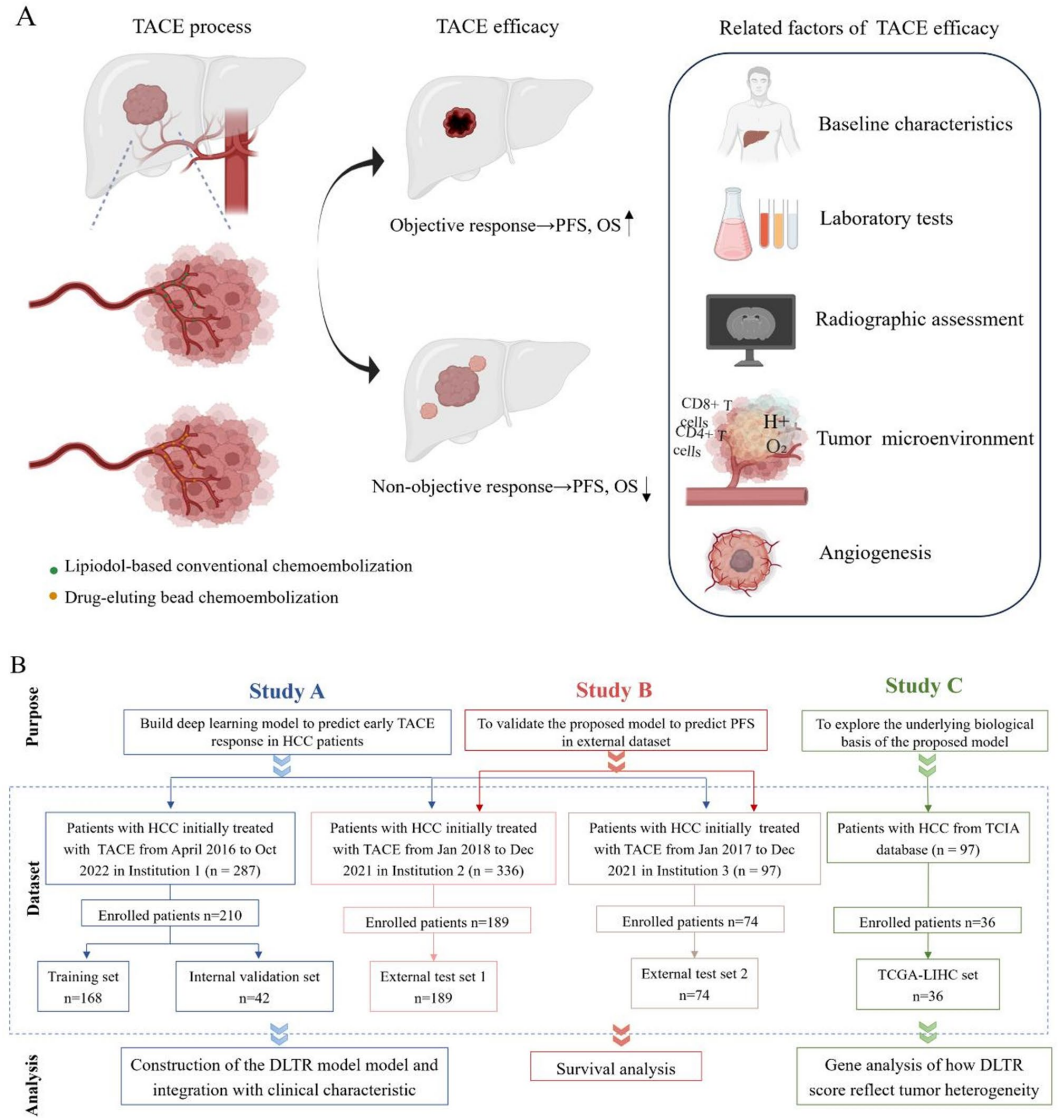
Factors related to TACE efficacy (**A**) and overall study design (**B**) illustrating the process of patient selection from three medical institutions and the Cancer Image Archive (TCIA) and downstream analysis. TACE, Transarterial chemoembolization; PFS, progression-free survival; OS, overall survival; HCC, hepatocellular carcinoma; DLTR, deep learning model for early assessment of TACE response; TCGA-LIHC, The Cancer Genome Atlas Liver Hepatocellular Carcinoma

Baseline characteristics, imaging evaluation, tumour, and its microenvironment heterogeneity are closely related to the efficacy of TACE, thereby affecting the prognosis of HCC patients (see Fig. 1 A). Owing to the lack of tissue specimens, the relationship between tumour biological information and TACE efficacy remains to be explored. Therefore, to systematically investigate these influence factors, the research process is divided into three parts (see Fig. 1B). First, a deep learning model that combines imaging and clinical indicators was constructed to predict response to TACE. Second, the correlation between the proposed model and the prognosis of HCC patients was analysed. Third, genetic analysis was performed to explore the biological functions associated with TACE outcomes.

Data were collected from consecutive patients with HCC at three hospitals. After applying the inclusion and exclusion criteria (see Appendix S1), a total of 473 cases were included, and the study flowchart is shown in Fig. 1B. Patients were divided into four cohorts: a training set (*n* = 168), an internal validation set (*n* = 42), and two external test sets (*n* = 189 and *n* = 74).

### TACE procedure and evaluation of TACE response

All included patients were treated with TACE, including conventional TACE and drug-eluting bead TACE (see Appendix S2). The objective response was determined by two experienced abdominal radiologists according to the post-operative MRI within 3 months. TACE response was performed according to the modified Response Evaluation Criteria in Solid Tumours (mRECIST) criterion [17]. In brief, the therapeutic response of initial TACE was classified into four grades, including complete response, partial response, progressive disease, and stable disease. Complete response and partial response patients were categorized as the objective response (OR) group, while progressive disease and stable disease patients as non-objective response (NOR) group.

### Development of DLTR for TACE response prediction

**Fig. 2:**
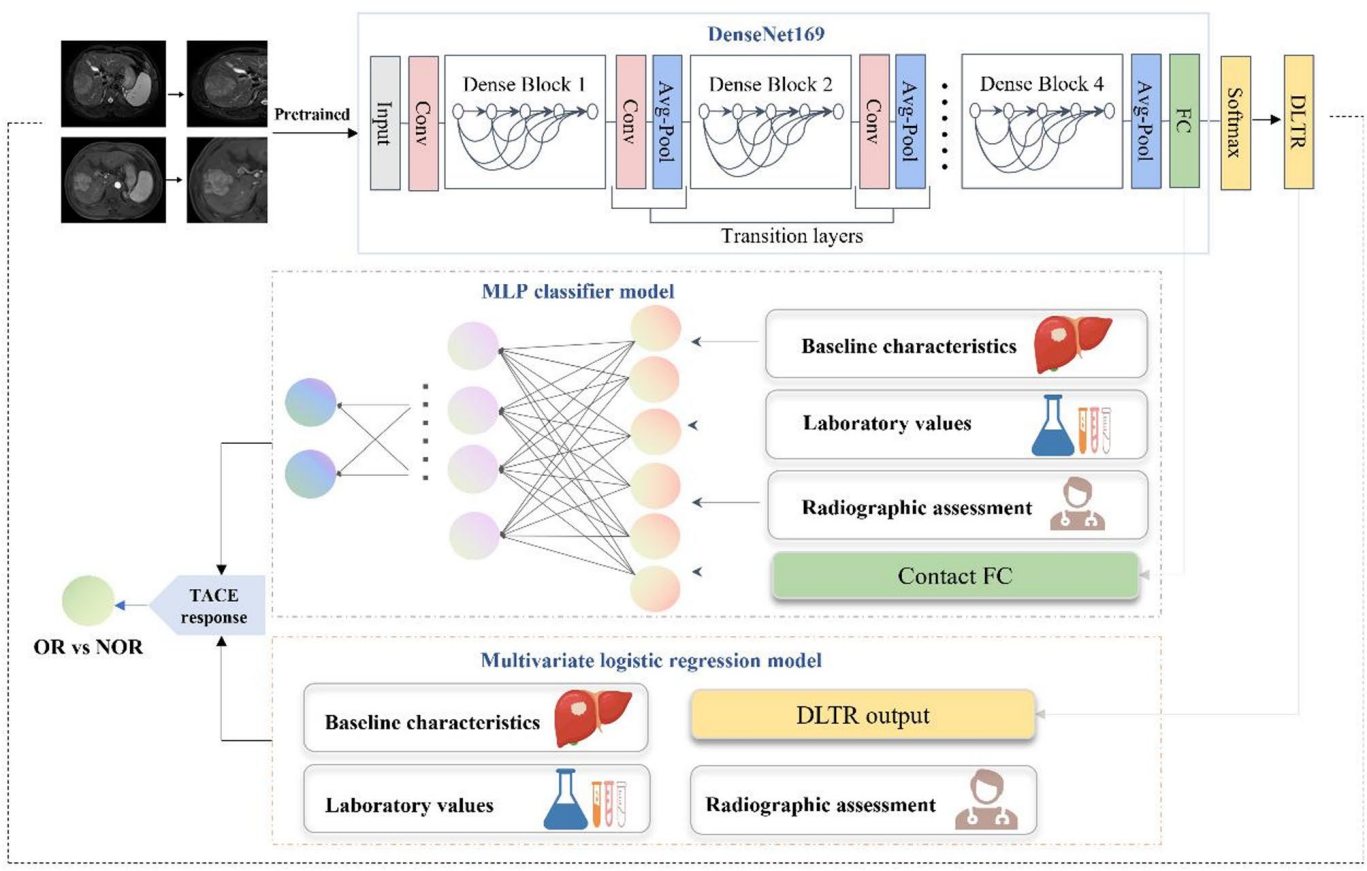
Workflow for deep learning model building based on MRI images and multi-information integration model to assess TACE outcomes in HCC. FC, fully connected layer; DLTR, deep learning model for early assessment of TACE response; TACE, Transarterial chemoembolization; OR, objective response; NOR, non-objective response

MRI examination and images preprocessing were illustrated in Appendix S3 and S4. CNNs can directly process medical images by passing them through multiple layers to extract important features and learn key information to identify and classify objects within the images. In the pre-experiment, four different CNNs, including Residual Network 18, Residual Network 50, DenseNet169, and Swin transformer [18–20], were constructed and trained to predict the efficacy of TACE, and the best-performing model was selected. Finally, DenseNet169 was presented as the most robust one in the pre-experiment. Therefore, the DenseNet169 algorithm was applied to construct the DLTR model, as illustrated in Fig. 2. DenseNet169 consists of four stacked dense blocks, with densely connected layers within each block, utilizing the BN + ReLU + Conv structure. Detailed information for the DenseNet169 setting is described in Appendix S5.

### Integration of DLTR and clinical factors

Clinical data were gathered from medical records, which included baseline characteristics, laboratory values, and radiographic assessment by two radiologists. As shown in Fig. 2, two models were developed to integrate DLTR and potential clinical factors efficiently. First, a multilayer perceptron (MLP) classifier was trained to predict the efficacy of TACE from the DLTR framework’s contact fully connected layer with other clinical indexes in a parallel way (hereafter, DLTR_MLP_). The framework of MLP was described in Appendix 6. Second, a multivariable logistic regression analysis was derived from the output of DLTR and other independent clinical factors (hereafter, DLTR_LR_). These included factors were screened through univariate analysis (*P*<0.05) in the training set and internal validation set.

### Survival analysis and biological basis exploration

Patients were divided into high-score and low-score groups based on the mean value of the DLTR_MLP_ score in 2 external test sets. The log-rank test was used to compare progression-free survival and overall survival by low or high-score group. progression-free survival was defined as the time interval between initial TACE to the date of progression, death, or censored at the date of the last follow-up (October 2023), while overall survival was defined as the time interval between initial TACE to the date of death, or censored at the date of the last follow-up (October 2023). Since the proposed model was established based on a local centre, survival analysis was performed in two external test sets. All patients were followed up by a telephone or digital medical record system. Biological basis exploration related to TACE outcomes was detailed in Appendix 7.

### Statistical analysis

The continuous baseline characteristics were analysed using variance analysis or the Kruskal-Wallis H test; the category data were analysed using the chi-square test or Fisher exact analysis. Area under the receiver operating characteristic (AUC), accuracy, sensitivity, specificity, positive predictive value, and negative predictive value were used to evaluate the performance of the model, and the probability threshold utilized for the accuracy outcomes remained consistent with the conventional standard of 0.5. All analyses were performed in SPSS, R, and Python v3.7.6, and a bilateral *P* < 0.05 was considered significant. The inference code of the proposed model can be accessed on https://github.com/wen-alan/TACE_response.

## Results

### Patient characteristics

The baseline information is outlined in Table 1. A total of 473 patients were included in this study, and divided into the training set, internal validation set, external test set 1, and external test set 2 (168, 42, 189, and 74 patients, respectively). The mean age of the entire cohort was 60 years; 85.84% (*n* = 406) of patients were male. There were 343 (72.52%) patients underwent conventional TACE and 130 (27.48%) patients treated with drug-eluting bead TACE. In terms of Child-Pugh classification, most patients were evaluated as class A (*n* = 356, 75.26%). In the assessment of cirrhosis, most patients (*n* = 308, 65.12%) presented with liver cirrhosis. Univariable analysis results of the training and internal validation sets are shown in Table S1. The results showed that ALT, Ascites, BCLC stage and portal venous invasion were significantly different between NOR and OR groups. Subsequently, these factors were submitted to construct a conventional clinical model via multivariable logistic regression analysis.

**Table 1:** Clinical characteristics of all included patients

	Total (*n* = 473)	Training set (*n* = 168)		Internal validation set (*n* = 42)	External test set 1 (*n* = 189)	External test set 2 (*n* = 74)	*P* value
Baseline characteristics							
Age (years)	60.38 ± 11.21		60.74 ± 11.39	59.62 ± 11.38	59.08 ± 11.17	63.34 ± 10.39	0.044^a#^
Sex, n (%)							0.926
Male	406 (85.84)		145 (86.31)	37 (88.10)	162 (85.71)	62 (83.78)	
Female	67 (14.16)		23 (13.69)	5 (11.90)	27 (14.29)	12 (16.22)	
Hypertension, n (%)							0.012
No	374 (79.07)		146 (86.90)	33 (78.57)	143 (75.66)	52 (70.27)	
Yes	99 (20.93)		22 (13.10)	9 (21.43)	46 (24.34)	22 (29.73)	
Diabetes, n (%)							0.013
No	424 (89.64)		160 (95.24)	36 (85.71)	167 (88.36)	61 (82.43)	
Yes	49 (10.36)		8 (4.76)	6 (14.29)	22 (11.64)	13 (17.57)	
HBsAg, n (%)							< 0.001
Negative	115 (24.31)		32 (19.05)	8 (19.05)	43 (22.75)	32 (43.24)	
Positive	358 (75.69)		136 (80.95)	34 (80.95)	146 (77.25)	42 (56.76)	
Child-Pugh classification, n (%)							0.003
A	356 (75.26)		121 (72.02)	32 (76.19)	157 (83.07)	46 (62.16)	
B	117 (24.74)		47 (27.98)	10 (23.81)	32 (16.93)	28 (37.84)	
BCLC stage, n (%)							< 0.001
A	184 (38.9)		82 (48.81)	19 (45.24)	53 (28.04)	30 (40.54)	
B/C	289 (61.1)		86 (51.19)	23 (54.76)	136 (71.96)	44 (59.46)	
Surgical methods, n (%)							< 0.001
c-TACE	343 (72.52)		112 (66.67)	28 (66.67)	131 (69.31)	72 (97.30)	
DEB‑TACE	130 (27.48)		56 (33.33)	14 (33.33)	58 (30.69)	2 (2.70)	
Laboratory tests							
AFP, n (%)							0.916
≤ 400 (ng/ml)	342 (72.3)		120 (71.43)	30 (71.43)	136 (71.96)	56 (75.68)	
>400 (ng/ml)	131 (27.7)		48 (28.57)	12 (28.57)	53 (28.04)	18 (24.32)	
CEA, n (%)							0.832
≤ 5ng༏ml	395 (83.51)		142 (84.52)	33 (78.57)	158 (83.60)	62 (83.78)	
>5ng༏ml	78 (16.49)		26 (15.48)	9 (21.43)	31 (16.40)	12 (16.22)	
AST, n (%)							0.663
≤ 40(U/L)	253 (53.49)		85 (50.60)	21 (50.00)	104 (55.03)	43 (58.11)	
>40(U/L)	220 (46.51)		83 (49.40)	21 (50.00)	85 (44.97)	31 (41.89)	
ALT, n (%)							0.005
≤ 50(U/L)	348 (73.57)		110 (65.48)	28 (66.67)	154 (81.48)	56 (75.68)	
>50(U/L)	125 (26.43)		58 (34.52)	14 (33.33)	35 (18.52)	18 (24.32)	
Albumin, n (%)							< 0.001
>40(g/L)	154 (32.56)		32 (19.05)	5 (11.90)	100 (52.91)	17 (22.97)	
≤ 40(g/L)	319 (67.44)		136 (80.95)	37 (88.10)	89 (47.09)	57 (77.03)	
Total bilirubin, n (%)							0.012
≤ 17.1(µmol/L)	250 (52.85)		74 (44.05)	21 (50.00)	116 (61.38)	39 (52.70)	
>17.1(µmol/L)	223 (47.15)		94 (55.95)	21 (50.00)	73 (38.62)	35 (47.30)	
Prothrombin time, n (%)							0.016
≤ 13(s)	319 (67.44)		100 (59.52)	28 (66.67)	132 (69.84)	59 (79.73)	
>13(s)	154 (32.56)		68 (40.48)	14 (33.33)	57 (30.16)	15 (20.27)	
Platelet count, n (%)							0.355
≥ 125 × 109/L	258 (54.55)		87 (51.79)	23 (54.76)	112 (59.26)	36 (48.65)	
<125 × 109/L	215 (45.45)		81 (48.21)	19 (45.24)	77 (40.74)	38 (51.35)	
NLR	2.88 (1.80–4.82)		2.94 (1.85–5.09)	3.91 (2.28–5.86)	2.77 (1.78–4.33)	2.97 (1.65–4.39)	0.229^b*^
PLR	107.14 (74.14–160.00)		106.46 (70.75–155.42)	111.39 (81.46–180.79)	107.14 (77.64–159.46)	107.02 (78.47–156.07)	0.836^b*^
Radiographic assessment							
Cirrhosis of background, n (%)							0.229
Absent	165 (34.88)		63 (37.50)	11 (26.19)	60 (31.75)	31 (41.89)	
Present	308 (65.12)		105 (62.50)	31 (73.81)	129 (68.25)	43 (58.11)	
Ascites, n (%)							0.328
Absent	365 (77.17)		136 (80.95)	30 (71.43)	146 (77.25)	53 (71.62)	
Present	108 (22.83)		32 (19.05)	12 (28.57)	43 (22.75)	21 (28.38)	
Tumour number, n (%)							< 0.001
Solitary	142 (30.02)		76 (45.24)	18 (42.86)	27 (14.29)	21 (28.38)	
Multiple	331 (69.98)		92 (54.76)	24 (57.14)	162 (85.71)	53 (71.62)	
Tumour diameter	5.00 (2.80–8.00)		4.30 (2.38–7.73)	4.90 (1.72–9.20)	5.40 (3.40–9.00)	4.60 (2.92–7.18)	0.053^b*^
Tumour margin, n (%)							< 0.001
Smooth margin	183 (38.69)		82 (48.81)	21 (50.00)	57 (30.16)	23 (31.08)	
Non-smooth margin	290 (61.31)		86 (51.19)	21 (50.00)	132 (69.84)	51 (68.92)	
Portal venous invasion, n (%)							0.863
Negative	375 (79.28)		132 (78.57)	32 (76.19)	150 (79.37)	61 (82.43)	
Positive	98 (20.72)		36 (21.43)	10 (23.81)	39 (20.63)	13 (17.57)	

Unless indicated otherwise, data are shown as number of patients, with the percentage in parentheses

*HBsAg* hepatitis B surface antigen, *BCLC* Barcelona Clinic Liver Cancer, *AFP* alpha-fetoprotein, *CEA* carcinoembryonic antigen, *AST* aspartate transaminase, *ALT* alanine transaminase, *NLR* neutrophils/lymphocytes ratio, *PLR* platelet/lymphocytes ratio

^a^ variance analysis

^b^ Kruskal-Wallis test; others (chi-square test or Fisher exact test)

# Data are mean ± standard deviation

* Data are medians, with interquartile ranges in parentheses

### Performance and validation of DLTR

**Fig. 3:**
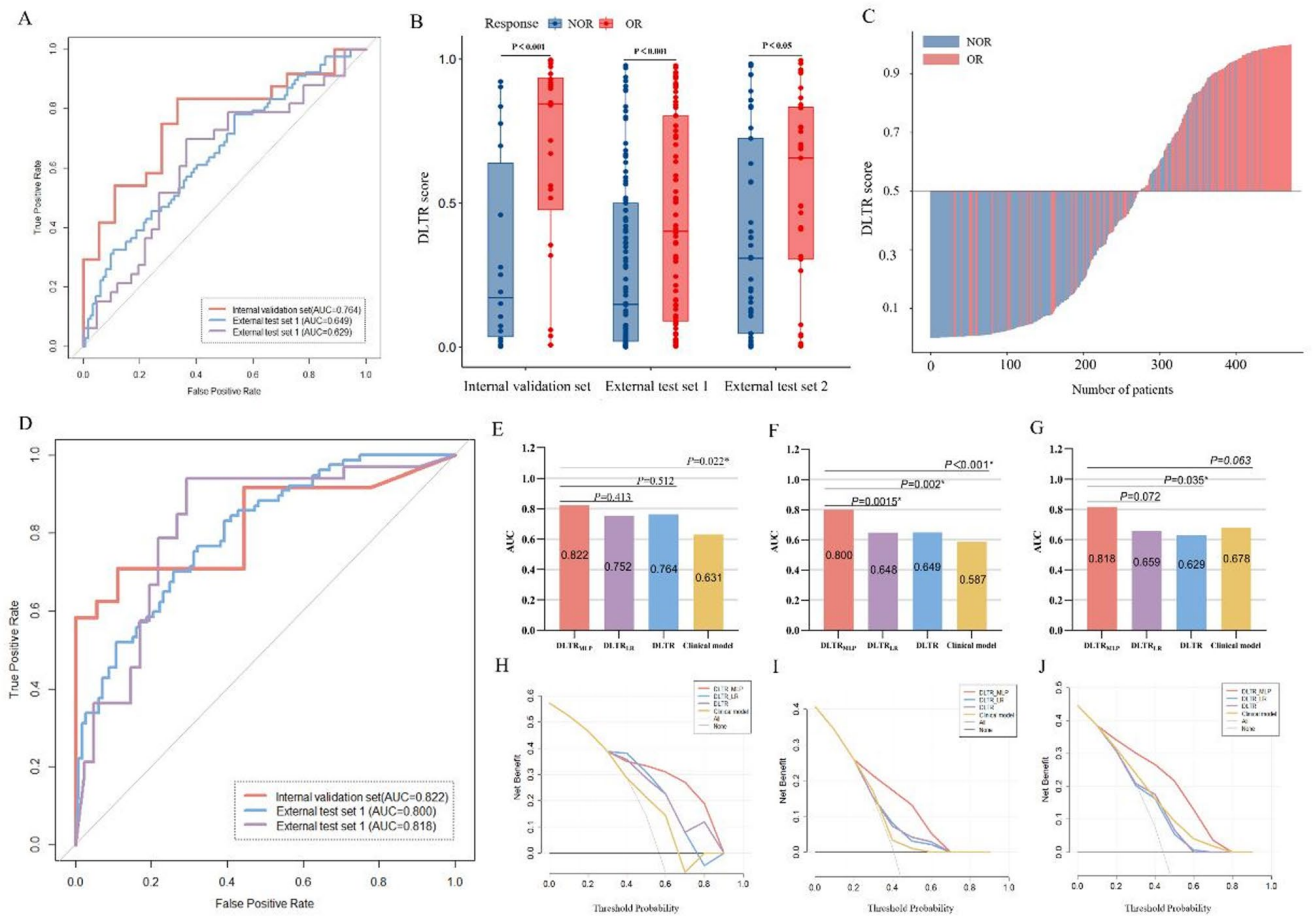
The performance of the DLTR and DLTR_MLP_ model for predicting the response to TACE therapy. **A** The receiver operating characteristic curves of the DLTR model in three cohorts; (**B**) The box figure shows the distribution of the DLTR score between OR and NOR groups in three cohorts; (**C**) Waterfall plot for DLTR score in the whole population; (**D**) The receiver operating characteristic curves of the DLTR_MLP_ model in three cohorts; (**E**-**G**) DeLong test for AUCs among the DLTR_MLP_ model, DLTR_LR_ model, DLTR model, and clinical model in the internal validation set (**E**), the external test set 1(**F**) and the external test set 2(**G**), respectively; (**H**-**J**) Decision curves analysis for the four different models in the internal validation set (**H**), the external test set 1(**I**) and the external test set 2 (**J**), respectively. OR, objective response; NOR, non-objective response; AUC, area under the receiver operating characteristic curve

According to the results of the pre-experiment (Table S2), the DLTR model based on DenseNet169 shows the optimal performance among the four CNN algorithms (including DenseNet169, ResNet50, ResNet18, and Swin transformer). Therefore, DLTR was constructed based on DenseNet169. The AUCs of DLTR to distinguish TACE response were 0.764 (95% confidence interval [CI]: 0.617–0.911), 0.649 (95% CI: 0.570–0.729), and 0.629 (95% CI: 0.498–0.760) in the internal validation set and external validation set 1 and external validation set 2, respectively (Fig. 3A). In the external test set 1, the relevant accuracy, sensitivity, specificity, positive predictive value, and negative predictive value were 59.3%, 77.9%, 46.4%, 50%, and 75.4%, respectively. For the external test set 2, the pertinent accuracy, sensitivity, specificity, positive predictive value, and negative predictive value were 66.2%, 69.7%, 63.4%, 60.5%, and 72.2%, respectively. Patients in the OR group were associated with a significantly higher DLTR score than those with NOR in all three cohorts (Fig. 3B).

To assess the practical value of the DLTR model, we endeavoured to determine the score ranges that yielded more accurate and less accurate predictions. We derived the probability threshold of the deep learning score as 0.5. The waterfall plot, which arranges patients’ scores from smallest to largest, indicates that patients with a DLTR score higher than 0.5 were mostly in the OR group (Fig. 3C).

### Predictive performance of the integrated models

In the following results, we incorporated the MRI image-based deep learning model and clinical model via two methodologies. Specifically, we established the DLTR_LR_ model using logistic regression and the DLTR_MLP_ model using MLP classifier, respectively. As shown in Fig. 3D, the DLTR_MLP_ model achieved better performance than the DLTR_LR_ model in the internal validation set (AUC: 0.822, 95% CI: 0.693–0.950), the external test set 1 (AUC: 0.800, 95% CI: 0.738–0.862), and the external test set 2 (AUC: 0.818, 95% CI: 0.717–0.919). A significant difference was observed in the external test set 1 (*P* = 0.0015), but not in the internal validation set (*P* = 0.413) and external test set 2 (*P* = 0.072) (Fig. 3E–G). Delong test results for ROC improvements of DLTR_MLP_ compared to single-modality models in multiple cohorts were presented in Table S3. Specifically, In the external test set 1, the associated accuracy, sensitivity, specificity, positive predictive value, and negative predictive value of DLTR_MLP_ were 71.4%, 76.6%, 67.9%, 62.1%, and 80.9%, respectively. For the external test set 2, the corresponding accuracy, sensitivity, specificity, positive predictive value, and negative predictive value were 81.1%, 93.9%, 70.7%, 72.1%, and 93.5%, respectively. The detailed performance of diverse models is summarized in Table 2.

**Table 2:** Predictive performance of various models in the internal validation and external test sets for TACE response

	Internal validation set(*n* = 42)	External test set 1 (*n* = 189)	External test set 2 (*n* = 74)
Clinical model			
AUC	0.631 (0.455–0.807)	0.587 (0.506–0.668)	0.678 (0.559–0.798)
Accuracy	0.619 (0.608–0.63)	0.624 (0.622–0.627)	0.649 (0.643–0.655)
Sensitivity	0.500 (0.300–0.700)	0.364 (0.256–0.471)	0.545 (0.376–0.715)
Specificity	0.778 (0.586–0.970)	0.804 (0.730–0.877)	0.732 (0.596–0.867)
PPV	0.750(0.538–0.962)	0.560 (0.422–0.698)	0.621 (0.444–0.797)
NPV	0.538 (0.347–0.730)	0.647 (0.568–0.727)	0.667 (0.529–0.804)
DLTR			
AUC	0.764 (0.617–0.911)	0.649 (0.570–0.729)	0.629 (0.498–0.760)
Accuracy	0.762 (0.753–0.770)	0.593 (0.590–0.595)	0.662 (0.656–0.668)
Sensitivity	0.833 (0.684–0.982)	0.779 (0.687–0.872)	0.697 (0.540–0.854)
Specificity	0.667 (0.449–0.884)	0.464 (0.372–0.557)	0.634 (0.487–0.782)
PPV	0.769 (0.607–0.931)	0.500 (0.411–0.589)	0.605 (0.450–0.761)
NPV	0.750 (0.538–0.962)	0.754 (0.652–0.855)	0.722 (0.576–0.869)
Integrated model			
DLTR _LR_			
AUC	0.752 (0.597–0.908)	0.648 (0.568–0.727)	0.659 (0.532–0.787)
Accuracy	0.786 (0.778–0.794)	0.656 (0.654–0.658)	0.662 (0.656–0.668)
Sensitivity	0.833 (0.684–0.982)	0.364 (0.256–0.471)	0.727 (0.575–0.879)
Specificity	0.722 (0.515–0.929)	0.857 (0.792–0.922)	0.610 (0.460–0.759)
PPV	0.800 (0.643–0.957)	0.636 (0.494–0.779)	0.600 (0.448–0.752)
NPV	0.765 (0.563–0.966)	0.662 (0.585–0.739)	0.735 (0.587–0.884)
DLTR _MLP_			
AUC	0.822 (0.693–0.950)	0.800 (0.738–0.862)	0.818 (0.717–0.919)
Accuracy	0.786 (0.778–0.794)	0.714 (0.712–0.716)	0.811 (0.807–0.815)
Sensitivity	0.708 (0.526–0.890)	0.766 (0.672–0.861)	0.939 (0.858–1.000.858.000)
Specificity	0.889 (0.744–1.000.744.000)	0.679 (0.592–0.765)	0.707 (0.568–0.847)
PPV	0.895 (0.757–1.000.757.000)	0.621 (0.523–0.719)	0.721 (0.587–0.855)
NPV	0.696 (0.508–0.884)	0.809 (0.729–0.888)	0.935 (0.849–1.000.849.000)

*DLTR*_*LR*_ DLTR output integrated with potential clinical factors using multivariate logistic regression (ascites, portal venous invasion status, ALT, BCLC stage included) *DLTR*_*MLP*_ DLTR output integrated with potential clinical factors using multilayer perceptron classifier, *AUC* area under the receiver operating characteristic curve, *PPV* positive predictive value, *NPV* negative predictive value

In addition, employing DLTR_MLP_ to predict response to TACE yields more net benefits than treat-all or treat-none strategies. According to the results of the decision curves analysis, if the threshold probability was greater than 0.4, utilizing the DLTR_MLP_ to predict TACE outcomes attains more benefits than the DLTR_LR_ model, DLTR model, and clinical model (Fig. 3H-J).

### Survival analysis

**Fig. 4:**
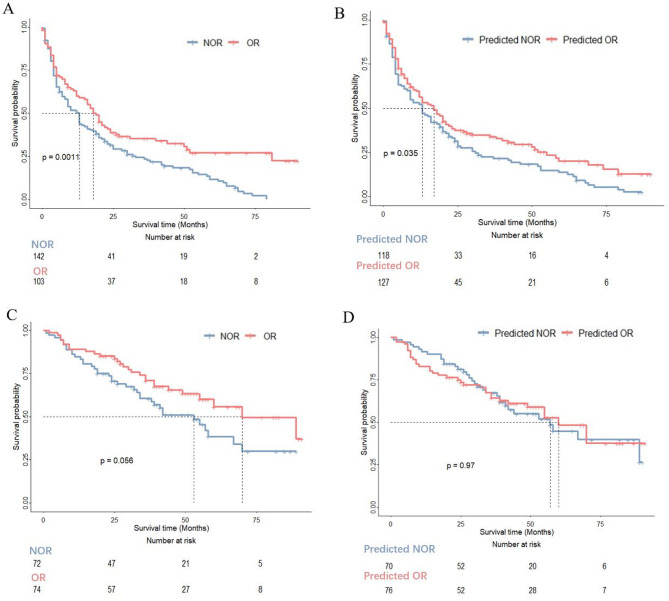
Survival curves according to mRECIST and model predicted determined tumour response status. **A**, **B** Progression-free survival curves and (**C**, **D**) overall survival curves determined by real tumour response status and the DLTR_MLP_ model predicted with Kaplan-Meier analysis. OR, objective response; NOR, non-objective response

The median follow-up time is 53 (interquartile range, 41–70) months. The median progression-free survival and overall survival of the whole external test cohorts were 14 (95% CI 10.5–15.7) months and 58 (95% CI 45.6–70.5) months, respectively. Particularly, progression-free survival according to the mRECIST criterion and DLTR_MLP_ model predicted OR and NOR are depicted in Fig. 4. The mRECIST criterion and DLTR_MLP_ were significantly associated with progression-free survival (*P* = 0.001 and *P* = 0.036, respectively) (Fig. 4A-B). Whereas the mRECIST criterion and DLTR_MLP_ were not significantly associated with overall survival (*P* = 0.056 and *P* = 0.97, respectively) (Fig. 4C-D).

### Biological basis exploration

**Fig. 5:**
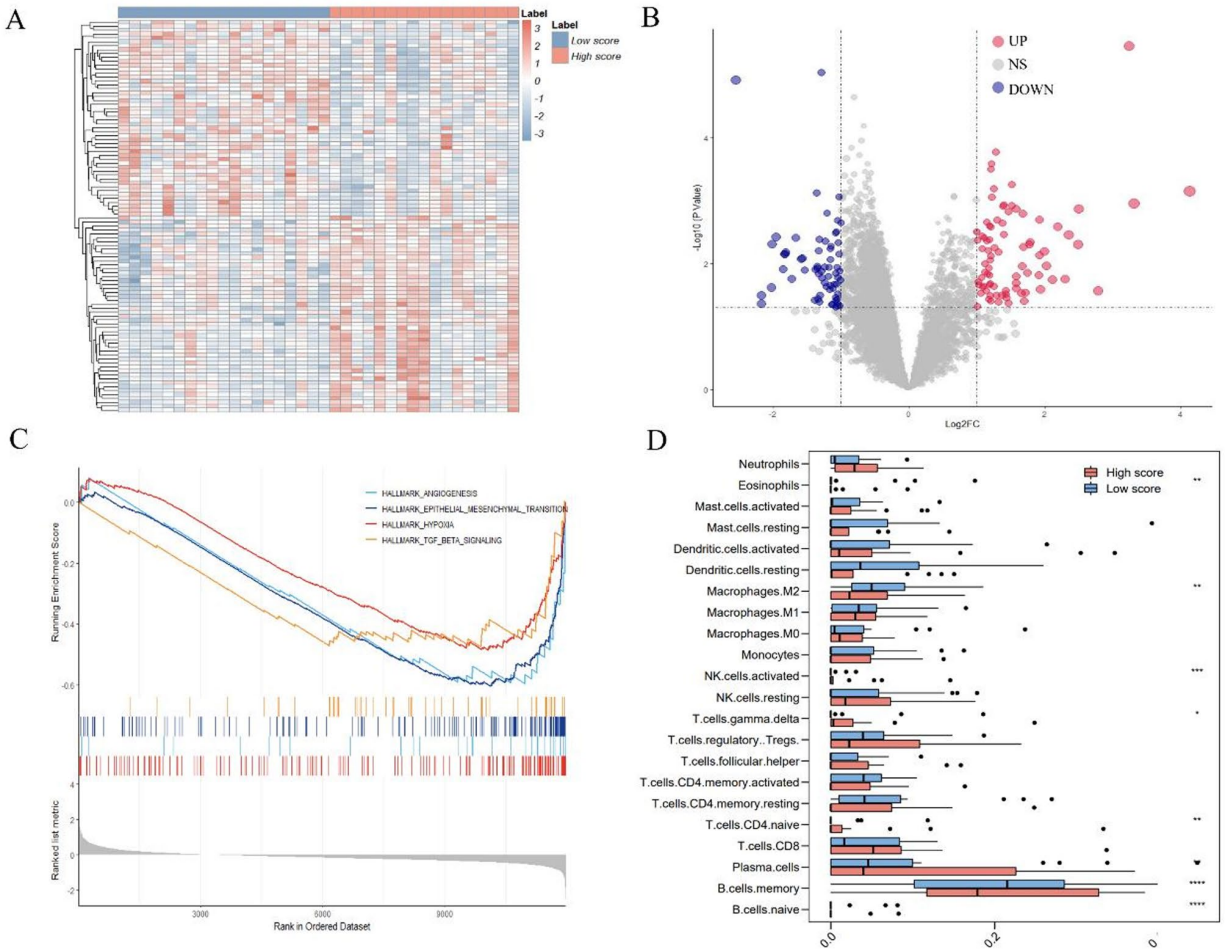
The genetic analysis for exploring the underlying biological basis of the developed DLTR model. **A** Heatmap of z-score normalized gene expressions presenting the differential expressed genes between high DLTR score group and low DLTR score group; (**B**) Volcano diagram of gene expression profiles in samples separated by high DLTR score versus low DLTR score. The red dots represent genes upregulated in patients categorized as high score, while the blue dots represent genes downregulated in patients categorized as low score. The x-axis denotes the fold change (log2 scale), whereas the y-axis indicates statistical significance (-log10 format); (**C**) Examples of the enrichment plot for the molecular pathways significantly associated with the DLTR; (**D**) Box plot depicting the estimation of the abundances of member cell types in a mixed cell population

As illustrated in Fig. 5A and B, significant differences were proved regarding gene expression between 17 high-score patients and 19 low-score patients from the TCIA dataset. The differentially expressed genes were mainly distinguished as two clusters, which were following divisions based on the DLTR score. A total of 149 differential expressed genes are listed in Appendix Excel. Further GSEA enrichment results exhibited that pathways promoting tumour proliferation and migration, such as angiogenesis, epithelial-mesenchymal transition, hypoxia, and TGF-β-Signalling, were significantly suppressed for tumours with a high DLTR score (Fig. 5C) [21–24]. In addition, tumours categorized as a high score exhibited less infiltrated M2 macrophage, but exhibited significantly more memory B cells than those categorized as a low score (Fig. 5D). Overall, these findings are consistent with their higher TACE response rates and favourable prognosis.

### Model explanation from CE-MRI images

**Fig. 6:**
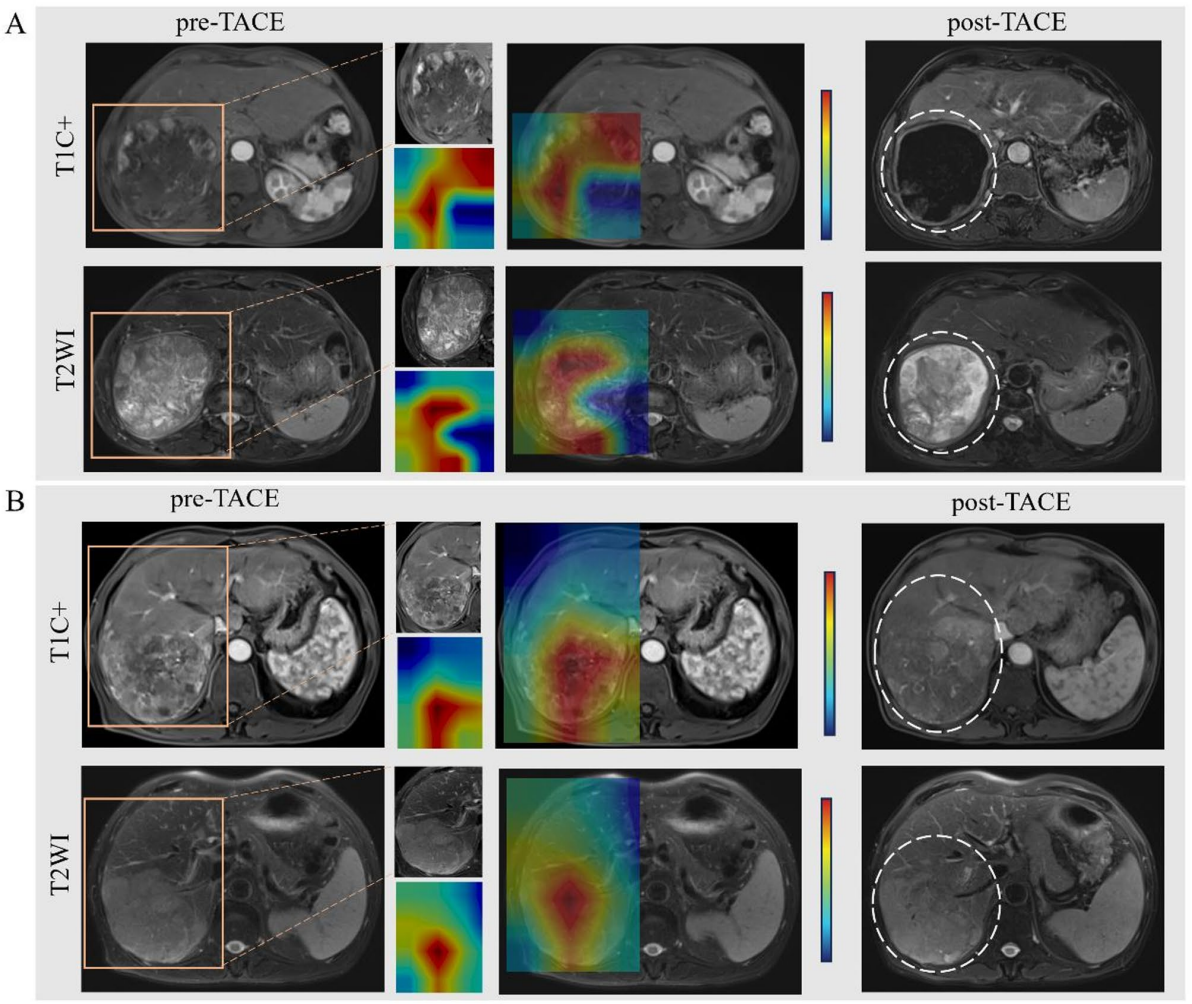
Representative cases of the attention map of (**A**) HCC with objective response to TACE, and(**B**) HCC with non-objective response to TACE. **A** A 72-year-old male HCC patient with a lesion diameter of 115 mm underwent MR scan 2 weeks before TACE. MR scan was re-examined 44 days after TACE. According to CE-MRI and T2WI images, the lesion diameter was similar to that before, but 95% necrosis occurred, so its TACE efficacy was evaluated as complete response (OR). **B** A 69-year-old male HCC patient, with a lesion diameter of 102 mm, underwent MR scan before (1 week) and after (31 days) TACE. The lesion diameter was stable and most of the lesions were viable. Hence, the corresponding early efficacy of TACE was evaluated as stable response (NOR)

After getting predictions from DLTR_MLP_, we employed class activation mapping attention map to make our method more transparent and highlight areas in the input liver radiographs that the network considers to be the most important [25]. In this way, class activation mapping can improve the interpretability of the CNN model. The attention map representative cases are shown in Fig. 6. On the T1C (hepatic arterial phase) attention map, the main activation area is the enhanced tumour area. On the T2 attention map, the main activation region involves the entire tumour region. In addition, the peritumoral region along the edge of the tumour is included in the activation region on the T1C and T2 saliency maps.

## Discussion

In this study, we constructed an MRI-based deep learning model (DLTR_MLP_) for early assessment of transarterial chemoembolization (TACE) response and efficiently integrated image information with relevant clinical factors using a multilayer perceptron (MLP) classifier in multicentre cohorts. The proposed DLTR_MLP_ model accurately predicted TACE response with area under the receiver operating characteristic curve (AUC) values of 0.8 and 0.818 for two external test sets. Additionally, our model outperformed other prediction models that relied on a single modality. Notably, the DLTR_MLP_ was found to be correlated with progression-free survival, offering valuable supplementary insights into the prognosis of patients with hepatocellular carcinoma (HCC). Genetic analysis showed that pathways that promote tumour proliferation and migration were significantly inhibited in tumours with high deep-learning scores.

TACE exhibits clinical diversity, requiring precise assessment of treatment response and prognosis prediction to select the most suitable treatment. Previous studies have identified various clinical characteristics and quantitative features derived from MRI images can predict TACE outcomes [26, 27]. However, accurately predicting responses to TACE for different individuals remains difficult in clinical practice. Deep learning can automatically and deeply explore and deconstruct information from the images, potentially improving the robustness of the model. For instance, She Y et al. put forth that a CT-based deep learning model could predict major pathological responses in patients with non-small cell lung cancer with an AUC of 0.75 [28]. In this study, we explored various CNNs and picked the best-performing one. The CNN we used, DenseNet-169 architecture, can enhance performance by retaining the low-level features in the images [19]. Our results suggest that the DLTR can initially stratify patients into OR and NOR groups, with those having high DLTR scores more likely to respond favourably. The primary roadblock of the deep learning model is its inability to be explained which can be problematic when implementing and promoting this “black-box” technology in clinical settings. To explore the prediction process of the proposed model, we visualized the focus area of the algorithm (as shown in Fig. 6).

Tumour heterogeneity is increasingly recognized as a key factor influencing the therapeutic response of tumours [29]. We hypothesized that the DLTR score, based on MRI images, may reflect the heterogeneity of tumour and tumour microenvironment, quantify tumour viability, and thus predict the early response to TACE. Prior research has shown that tumour intrinsic characteristics significantly affect the efficacy of TACE treatments [30]. As such, we further explored the biological basis for the DLTR score. Enrichment analysis indicated that pathways associated with tumour proliferation and migration, such as angiogenesis, epithelial-mesenchymal transition, hypoxia, and TGF-β-Signalling, were notably down-regulated in the high DLTR score group [21–24]. In addition, tumours classified into high-score groups exhibited anti-tumour immune cell infiltration in the microenvironment. These findings suggested that deep learning provides valuable information that reflects the tumour heterogeneity and the tumour microenvironment, which can affect the sensitivity of TACE treatment. Furthermore, our observations support the notion that a high DLTR score is associated with a higher likelihood of favourable TACE outcomes.

We utilized two different methods, MLP classifier and logistic regression analysis, to integrate DLTR and other clinical factors. The results demonstrated that the MLP classifier outperformed LR in the internal validation set and two external test sets. The superior prediction performance of DLTR_MLP_ can be attributed to the effective integration of key deep learning features with clinical characteristics in an early fusion manner. Besides, we applied Dropout and Batch normalization to reduce overfitting. A study of Alzheimer’s disease classification also found that the MLP classifier with multi-scale inputs generalized well across four datasets [31]. Our DLTR_MLP_ exhibited superior predictive ability compared to single-modality models across all cohorts in the current study, including the clinical model and the DLTR model. This indicates that the integration of multimodal information can significantly improve the performance of the model. Moreover, we found that DLTR_MLP_ was significantly associated with progression-free survival in Patients with HCC (*P* < 0.05), consistent with the results of LIU et al. [32]. Patients with poor prognosis should choose other treatment strategies on time to avoid unnecessary adverse reactions and improve the survival prognosis of patients. By utilizing imaging to identify crucial tumour features and heterogeneity, our DLTR_MLP_ model can improve tumour characterization and treatment.

This study has several limitations. First, the retrospective nature of the data collection may introduce selection bias. To mitigate this concern, we included multi-center validation cohorts to strengthen the reliability of our findings. Second, the biological interpretation of the deep learning model remains preliminary. While we identified associations between MRI features and gene expression signatures, the underlying mechanisms linking specific biological processes to TACE response require further investigation. Third, our model provides predictions only at the patient level and does not account for potential response heterogeneity across individual lesions in patients with multifocal HCC. Future studies should explore lesion-level analysis by developing an advanced algorithm capable of segmenting and evaluating each tumour nodule separately. Fourth, the use of 2D axial MRI images may overlook valuable spatial information contained in volumetric data. We plan to improve model performance by incorporating 2.5D or 3D approaches in subsequent work. Finally, prospective validation in clinical practice is necessary to assess the real-world utility of our model. Next steps include conducting clinical trials to evaluate the impact of model-guided decisions on patient outcomes and developing a user-friendly interface for seamless integration into existing clinical workflows such as PACS.

In conclusion, we developed an MRI-based CNN model and incorporated clinical factors using a multilayer perceptron approach to enhance the prediction of early TACE response for individualized treatment. Moreover, the potential biological basis of deep learning scoring may be related to the pathways that mediate tumour proliferation.

## Supplementary Information

Supplementary Material 1.

## Data Availability

The inference code of the proposed model can be accessed on https://github.com/wen-alan/TACE/_response. Data is available upon reasonable request from the corresponding author.

## Abbreviations

ALT: Alanine aminotransferase
AP: Arterial phase
AST: Aspartate aminotransferase
AUC: Area under the receiver operating characteristic curve
BCLC: Barcelona clinic liver cancer staging
CI: Confidence interval
CNN: Convolutional neural networks
CT: Computed tomography
CE-MRI: Contrast-enhanced magnetic resonance imaging
DP: Delayed phase
DWI: Diffusion-weighted imaging
HbsAg: Hepatitis b surface antigen
HCC: Hepatocellular carcinoma
MLP: Multilayer perceptron
mRECIST: Modified response evaluation criteria in solid tumours
MRI: Magnetic resonance imaging
OS: Overall survival
PFS: Progression-free survival
PVP: Portal venous phase
ReLU: Rectified linear unit
ROC: Receiver operating characteristic curve
TACE: Transarterial chemoembolization
T2WI: T2-weighted imaging
