# Supplementary material for: MRI-based deep learning model for early TACE response prediction in HCC: multicenter validation with biological insights
Source: BMC Cancer. 2025 Nov 24;25:1810. doi: 10.1186/s12885-025-15273-8 (PMC12642030; doi:10.1186/s12885-025-15273-8)
Supplement: Supplementary file 1 — Supplementary Material 1. [file 12885_2025_15273_MOESM1_ESM.docx]

Part I. Supplementary Information

Part II. Supplementary Figures

Part III. Supplementary Tables

**Part I. Supplementary Information**

**Appendix S1: Inclusion and exclusion criteria**

Inclusion criteria are as follows:

(a) Patients with HCC who underwent initial transarterial chemoembolization (TACE) in institution 1 (April 1, 2016 to December 31, 2022), institution 2 (January 1, 2018 to December 31, 2021), and institution 3 (January 1, 2017 to December 31, 2021); HCC was confirmed by imaging examinations evaluation [1]. Biopsy was carried out if the nodule did not indicate typical features in medical images.

(b) Contrast-enhanced magnetic resonance imaging (CE-MRI) was performed before and after TACE;

(c) Relevant clinical information was available (i.e. demographics, preoperative hepatitis, serum alpha-fetoprotein (AFP) levels, and liver function tests).

Exclusion criteria are as follows:

(a) Underage patients;

(b) Synchronous therapies during follow-up time, such as resection, and systemic chemotherapy;

(c) With other malignancies and follow-up for less than 3 months after the operation.

(d) Very small region of interest (ROI) or patients with poor MRI image quality.

**Appendix S2: TACE Procedure**

All TACE treatment were administered by interventional physicians with at least 10 years of experience and approved by the patients. Interventional physicians choose conventional TACE (cTACE) or drug-eluting bead TACE (DEB-TACE) based on tumor burden and patient characteristics. The basic procedure for DEB-TACE is similar to cTACE, except for the embolic agent. cTACE uses lipiodol (Guerbet), gelatin sponge particles, and polyvinyl alcohol as embolic agents. Selective or super-selective embolization of the artery directly supplying the tumor was carried out whenever technically justified [2]. For DEB-TACE, 100-300μm diameter CalliSpheres® Beads (CB; Jiangsu Hengrui Pharmaceutical Co., Ltd.) were used as carriers, loaded with 60-80mg epirubicin, pirarubicin, or doxorubicin. After TACE therapy, all patients were admitted for postoperative supportive care and treated routinely.

**Appendix S3: MRI Image Acquisition**

**Zhejiang University Lishui Hospital**: All recruited patients underwent conventional and enhanced scan using 1.5-T and 3.0-T MR scanners.

For the Philips ENGENIA 3.0-T MR scanner (Philips Medical Systems), imaging sequences included axial T2 weighted imaging (T2WI) and enhanced-T1 high resolution isotropic volume examination (e-THRIVE). The image acquisition parameters were as follows: (1) T2WI, repetition time (TR) 3000 ms, echo time (TE) 200 ms, matrix: 200×195, thickness 7 mm, spacing 1mm; (2) e-THRIVE, TR 2.5 ms, TE 2.9 ms, matrix 289 × 192, thickness 3 mm, spacing 0.5 mm, FOV 38 cm × 38 cm. Gadopentetate dimeglumine (Gd-DTPA) (0.1 mmol/kg) was injected into the elbow vein at a flow rate of 2.5 ml/s by a high-pressure syringe, and then 15 ml of normal saline was injected at the same flow rate. The hepatic arterial phase (AP), portal venous phase (PVP), and delayed phase (DP) images were scanned in 10, 60, and 150 s, respectively.

For the German MAGNETOM Area 1.5T MR scanner, conventional MRI scan sequences included: (1) T2WI: TR 3500 ms, TE 90 ms, FOV 38 cm × 38 cm, matrix 320 × 320; (2) Enhanced scan was performed with three-dimensional volumetric interpolated breath-hold examination (3D-VIBE), TR 4.1 ms, TE 1.8 ms, FOV 38 cm × 38 cm, matrix 320 × 320. The contrast agent was Gd-DTPA, the dose was 0.1 mmol/kg, and the flow rate was 2 ml/s. The images of AP, PVP, and DP were collected at 25 s, 60 s and 180 s, respectively. The layer thickness of all sequences was 5 mm, and the layer spacing was 1 mm.

**The First Affiliated Hospital of Zhejiang University**: GE Signa HDXT 3.0T MR scanner and 8-channel phased array surface coil were used for liver MRI scan. Contrast-enhanced T1WI Liver Acquisition with Volume Acceleration (LAVA), TR 3.1 ms, TE 1.5 ms, reverse time 5.0 ms, reverse angle 15°, matrix 320 × 256, slice thickness 5 mm, no interval scanning, FOV 40 cm × 32 cm. Gd-DTPA (0.1 mmol/kg) was injected into the elbow vein at a flow rate of 2.5 ml/s by a high-pressure syringe, and then 15 ml of normal saline was injected at the same flow rate, and the scanning of AP, PVP, and DP was performed at 10 s, 60 s and 150 s, respectively.

**The Hangzhou First People’s Hospital**: The Philips Ingenia 3.0T MR scanner was used to collect images using a 16-channel abdominal phased array coil. MRI scan sequences included: (1) T2WI: TR 4064ms, TE 105ms, FOV 42cm × 42cm, matrix 320 × 320; (2) Enhanced scanning: e-THRIVE, TR 3.1 ms, TE 1.5 ms, FOV 40 cm × 36 cm, matrix 320 × 256, contrast agent Gd-DTPA, dose 0.1 mmol / kg, flow rate 2 ml / s. The images of AP, PVP, and DP were collected at 25 s, 60 s and 180 s. The layer thickness of all sequences was 5 mm and the layer spacing was 0 mm.

**Appendix S4: MRI examination and Images Preprocessing**

The input images were selected from the axial largest T2 weighted imaging (T2WI) and arterial phase (AP) images, respectively. Two experienced radiologists segmented the targeted lesion independently using a rectangular shape. Examples of the cropping process are illustrated in Figure S1. If the radiologists encounter disagreement, they would negotiate until they reach a consensus. Before being fed into the deep learning model, all images were uniformly resized to 128×128 pixels. Additionally, random cropping, brightness, and contrast data augmentation techniques were applied. Finally, all images were normalized using Z-Score to ensure consistent scaling across the dataset.

**Appendix S5: Neural Network Settings**

Medical images are less abundant compared to natural images, making CNNs well-suited for modeling with large datasets. Therefore, the concept of transfer learning was employed in the experiment. ImageNet [3], the massive natural image database, was used to pre-train the DLTR model, followed by fine-tuning with MRI images. By leveraging the abundant data from the ImageNet library, the CNN can achieve better results when processing TACE images, thereby addressing the challenge of limited MRI images data. To mitigate overfitting, Dropout was employed in all networks.

Transition layers in DenseNet169 are mainly used to connect two adjacent DenseBlocks. By integrating the features obtained by the previous DenseBlock, the width and height of the previous DenseBlock can be reduced, thereby achieving downsampling effect, halving the dimensions of the feature maps. The transition layer comprises a 1×1 convolution (used to adjust the number of channels) and 2×2 average pooling (used to reduce the size of the feature maps), effectively compressing the model. Compared to ResNet, DenseNet has fewer parameters, retains lower-level features for both propagation and prediction, strengthens feature reuse through skip connections. Hence, DenseNet is more resistant to issues such as gradient vanishing/exploding and network degradation.

We conducted modeling experiments on the PyTorch platform (version 1.10.0) with an NVIDIA 3090TI GPU. The Adam algorithm was used to optimize the network training process. Dropout probability in the network was set at 0.2, L2 regularization coefficient at 5e-4, and cosine annealing learning rate at 5e-4. A batch size of 32 was used during network training, and the models were trained for 20 epochs.

**Appendix S6: Multilayer Perceptron Framework**

The multilayer perceptron (MLP) architecture consists of multiple layers of artificial neural networks between input features and output predictions [4]. It provides an end-to-end modeling approach which is widely used in clinical feature modeling. In this study, the MLP network consists of 4 layers, with each layer connected through the ReLU activation function. Meanwhile, BatchNorm and Dropout modules are used to improve performance. BatchNorm is employed for batch normalization to prevent overfitting and accelerate training [5]. Besides, Dropout is utilized as a regularization tool to randomly deactivate neurons during training [6]. Furthermore, L2 regularization is employed to control model complexity and mitigate overfitting.

**Appendix S7: Biological basis exploration**

To explore the underlying biological basis of the DLTR, gene analyses were performed among patients with RNA-sequencing data from The Cancer Genome Atlas (TCGA) database. There were 97 Patients with HCC in The Cancer Imaging Archive (TCIA) database (TCGA-LIHC). Of these, 37 patients were excluded due to missing preoperative MRI, and 24 due to inferior image quality. A total of 36 patients with both available CE-MRI images and RNA-sequence data were included. Using the median value of the DLTR score, patients were stratified into 17 patients with a high DLTR score and 19 patients with a low DLTR score. The R package limma was utilized to recognize differential expression genes (DEGs) between these two groups based on the criteria of logFC >1 and adjusted *P* value＜0.05. Then, gene set enrichment analysis (GSEA) was carried out using the R package clusterProfiler to identify the signaling pathways related to the DLTR score [7]. Immune microenvironment analyses were performed through CibersortX (https://cibersortx.stanford.edu) to evaluate the abundance of member cell types in a mixed cell population.

**Reference**

1 Zhou J, Sun H, Wang Z, Cong W, Wang J, Zeng M, et al. Guidelines for the Diagnosis and Treatment of Hepatocellular Carcinoma (2019 Edition). Liver Cancer. 2020 Dec;9(6):682–720.

2 Golfieri R, Cappelli A, Cucchetti A, Piscaglia F, Carpenzano M, Peri E, et al. Efficacy of selective transarterial chemoembolization in inducing tumor necrosis in small (<5 cm) hepatocellular carcinomas. Hepatology. 2011 May;53(5):1580–9.

3 Krizhevsky A, Sutskever I, Hinton GE. Imagenet classification with deep convolutional neural networks. Advances in neural information processing systems. 2012 [cited 2024 Feb 10]. ;25. Available from: https://proceedings.neurips.cc/paper/2012/hash/c399862d3b9d6b76c8436e924a68c45b-Abstract.html

4 Kruse R, Mostaghim S, Borgelt C, Braune C, Steinbrecher M. Multi-layer Perceptrons. Computational Intelligence. Cham; 2022; pp 53–124.

5 Ioffe S, Szegedy C. Batch normalization: Accelerating deep network training by reducing internal covariate shift. International conference on machine learning. pmlr; 2015; [cited 2024 Feb 10]; pp 448–56.

6 Srivastava N, Hinton G, Krizhevsky A, Sutskever I, Salakhutdinov R. Dropout: a simple way to prevent neural networks from overfitting. The journal of machine learning research. 2014;15(1):1929–58.

7 Yu G, Wang L-G, Han Y, He Q-Y. clusterProfiler: an R Package for Comparing Biological Themes Among Gene Clusters. OMICS: A Journal of Integrative Biology. 2012 May;16(5):284–7.

**Part II. Supplementary Figures**


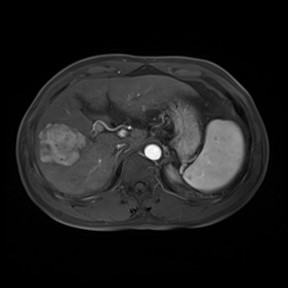

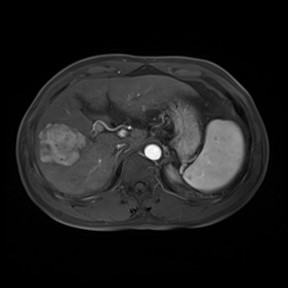

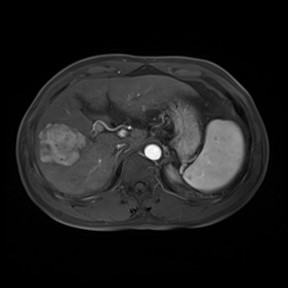

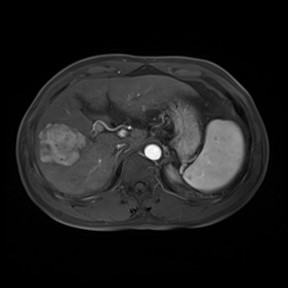

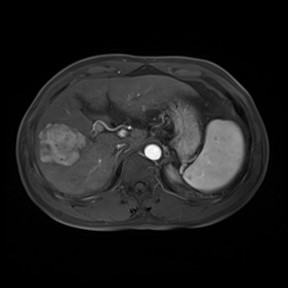

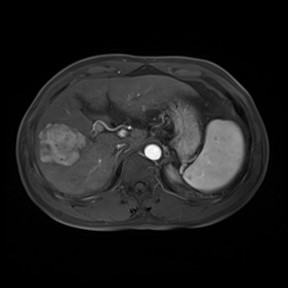

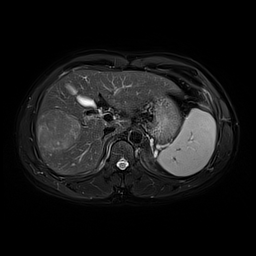

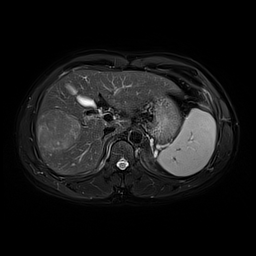

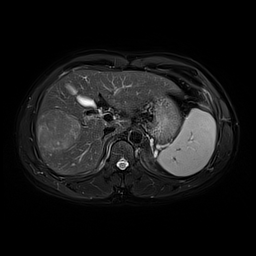

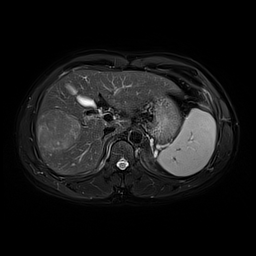

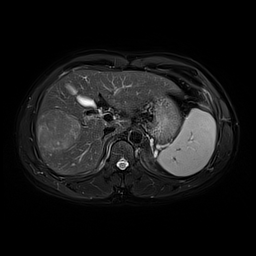

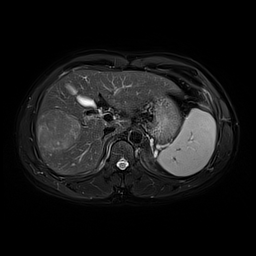


Figure S1. The ROIs cropped by radiologists.

**Figure S2. ROC curve of different models in (a) Internal validation set; (b) External test set 1; (c) External test set 2**


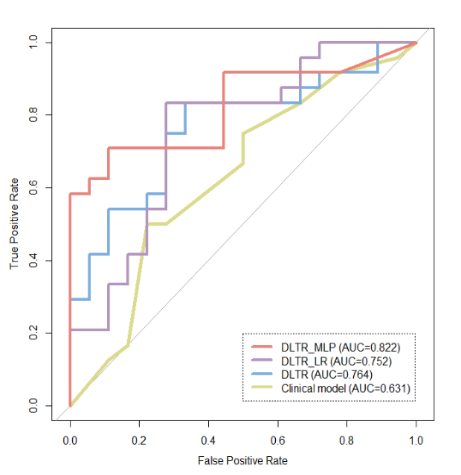

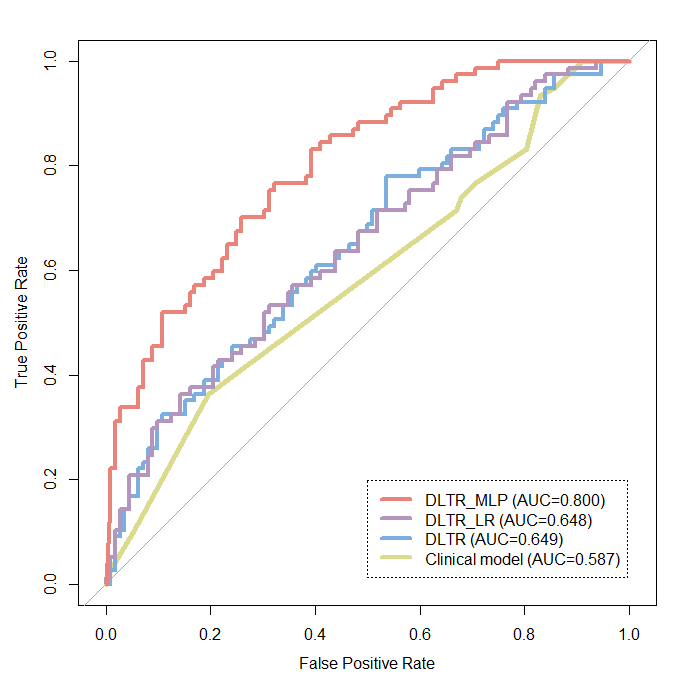

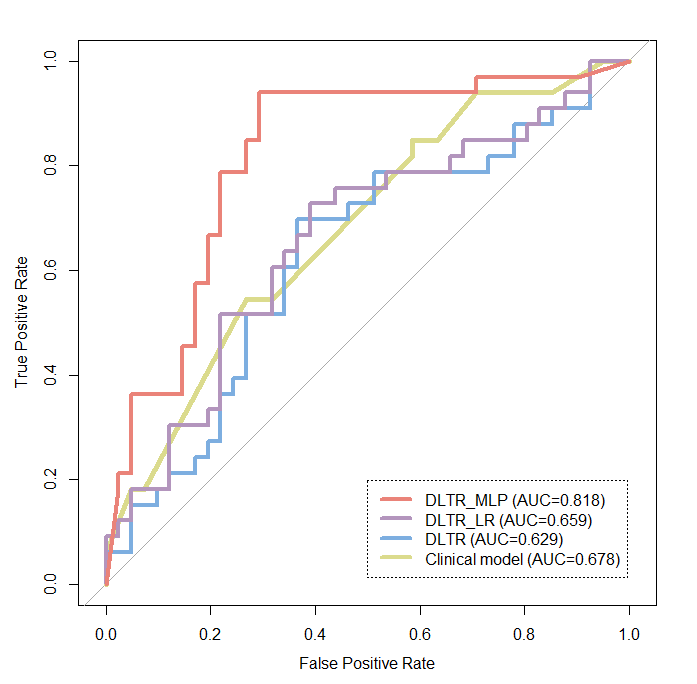


a

b

c

**Part III. Supplementary Tables**

**Table S1.** **Univariable analysis for the training and internal validation sets**

|  | Training set (n=168) | | *P* value | Internal validation set (n=42) | | *P* value |
| --- | --- | --- | --- | --- | --- | --- |
|  | NOR (n=87) | OR (n=81) |  | NOR (n=18) | OR (n=24) |  |
| Age (years) | 59.45±12.12 | 62.14±10.46 | 0.127^a#^ | 58.17±11.429 | 60.71±11.46 | 0.581^a#^ |
| Sex, n (%) |  |  | 0.682 |  |  | 0.074 |
| Male | 76(87.4) | 69(85.2) |  | 14(77.8) | 23(95.8) |  |
| Female | 11(12.6) | 12(14.8) |  | 4(22.2) | 1(4.2) |  |
| Hypertension, n (%) |  |  | 0.273 |  |  | 1 |
| No | 78(89.7) | 68(84.0) |  | 14(77.8) | 19(79.2) |  |
| Yes | 9(10.3) | 13(16.0) |  | 4(22.2) | 5(20.8) |  |
| Diabetes, n (%) |  |  | 0.407 |  |  | 0.068 |
| No | 84(96.6) | 76(93.8) |  | 13(72.2) | 23(95.8) |  |
| Yes | 3(3.4) | 5(6.2) |  | 5(27.8) | 1(4.2) |  |
| HBsAg, n (%) |  |  | 0.574 |  |  | 0.256 |
| Negative | 18(20.7) | 14(17.3) |  | 5(27.8) | 3(12.5) |  |
| Positive | 69(79.3) | 67(82.7) |  | 13(72.2) | 21(87.5) |  |
| Child-Pugh classification, n (%) |  |  | 0.052 |  |  | 0.72 |
| A | 57(65.5) | 64(79.0) |  | 13(72.2) | 19(79.2) |  |
| B | 30(34.5) | 17(21.0) |  | 5(27.8) | 5(20.8) |  |
| BCLC stage, n (%) |  |  | 0.168 |  |  | **0.049** |
| A | 38(43.7) | 44(54.3) |  | 5(27.8) | 14(58.3) |  |
| B/C | 49(56.3) | 37(45.7) |  | 13(72.2) | 10(41.7) |  |
| Surgical methods, n (%) |  |  | 0.512 |  |  | 1 |
| c-TACE | 60(69.0) | 52(64.2) |  | 12(66.7) | 16(66.7) |  |
| DEB‑TACE | 27(31.0) | 29(35.8) |  | 6(33.3) | 8(33.3) |  |
| **Laboratory tests** |  |  |  |  |  |  |
| AFP, n (%) |  |  | 0.961 |  |  | 0.921 |
| ≤400 (ng/ml) | 62(71.3) | 58(71.6) |  | 13(72.2) | 17(70.8) |  |
| ＞400 (ng/ml) | 25(28.7) | 23(28.4) |  | 5(27.8) | 7(29.2) |  |
| CEA, n (%) |  |  | 0.532 |  |  | 0.462 |
| ≤5ng／ml | 75(86.2) | 67(82.7) |  | 13(72.2) | 20(83.3) |  |
| ＞5ng／ml | 12(13.8) | 14(17.3) |  | 5(27.8) | 4(16.7) |  |
| AST, n (%) |  |  | 0.533 |  |  | 0.061 |
| ≤40(U/L) | 42(48.3) | 43(53.1) |  | 6(33.3) | 15(62.5) |  |
| ＞40(U/L) | 45(51.7) | 38(46.9) |  | 12(66.7) | 9(37.5) |  |
| ALT, n (%) |  |  | **0.049** |  |  | 0.53 |
| ≤50(U/L) | 63(72.4) | 47(58.0) |  | 11(61.1) | 17(70.8) |  |
| ＞50(U/L) | 24(27.6) | 34(42.0) |  | 7(38.9) | 7(29.2) |  |
| Albumin, n (%) |  |  | 0.574 |  |  | 1 |
| ＞40(g/L) | 18(20.7) | 14(17.3) |  | 2(11.1) | 3(12.5) |  |
| ≤40(g/L) | 69(79.3) | 67(82.7) |  | 16(88.9) | 21(87.5) |  |
| Total bilirubin, n (%) |  |  | 0.92 |  |  | 0.533 |
| ≤17.1(µmol/L) | 38(43.7) | 36(44.4) |  | 10(55.6) | 11(45.8) |  |
| ＞17.1(µmol/L) | 49(56.3) | 45(55.6) |  | 8(44.4) | 13(54.2) |  |
| Prothrombin time, n (%) |  |  | 0.805 |  |  | 0.508 |
| ≤13(s) | 51(58.6) | 49(60.5) |  | 11(61.1) | 17(70.8) |  |
| ＞13(s) | 36(41.4) | 32(39.5) |  | 7(38.9) | 7(29.2) |  |
| Platelet count, n (%) |  |  | 0.77 |  |  | 0.245 |
| ≥125×109/L | 46(52.9) | 41(50.6) |  | 8(44.4) | 15(62.5) |  |
| ＜125×109/L | 41(47.1) | 40(49.4) |  | 10(55.6) | 9(37.5) |  |
| NLR | 3.23(1.85 - 5.07) | 2.84(1.68-5.26) | 0.770^b*^ | 3(2.17-5.76) | 4.66(2.23 - 6.20) | 0.485^b*^ |
| PLR | 109.23(75.7 - 171.54) | 105.71(64.83 - 147.64) | 0.167^b*^ | 126.80±61.39 | 126.80±61.39 | 0.837^a*^ |
| **MR imaging features** |  |  |  |  |  |  |
| Cirrhosis of background, n (%) |  |  | 0.604 |  |  | 0.483 |
| Absent | 31(35.6) | 32(39.5) |  | 6(33.3) | 12(50.0) |  |
| Present | 56(64.4) | 49(60.5) |  | 12(66.7) | 12(50.0) |  |
| Ascites, n (%) |  |  | **0.011** |  |  | 0.554 |
| Absent | 64(73.6) | 72(88.9) |  | 12(66.7) | 18(75.0) |  |
| Present | 23(26.4) | 9(11.1) |  | 6(33.3) | 6(25.0) |  |
| Tumor number, n (%) |  |  | 0.842 |  |  | 0.28 |
| Solitary | 40(46.0) | 36(44.4) |  | 6(37.5) | 7(53.8) |  |
| Multiple | 47(54.0) | 45(55.6) |  | 10(62.5) | 6(46.2) |  |
| Tumor diameter | 4.9(2.7 - 9. 2) | 4.1(2.2 - 7.3) | 0.100 ^b*^ | 7.8(1.75 - 10.77) | 4.3(1.55 - 6.13) | 0.101^b*^ |
| Tumor margin, n (%) |  |  | 0.433 |  |  | 0.212 |
| Smooth margin | 45(51.7) | 37(45.7) |  | 7(38.9) | 14(58.3) |  |
| Non-smooth margin | 42(48.3) | 44(54.3) |  | 11(61.1) | 10(41.7) |  |
| Portal venous invasion, n (%) |  |  | **0.044** |  |  | 0.07 |
| Negative | 63(72.4) | 69(85.2) |  | 11(61.1) | 21(87.5) |  |
| Positive | 24(27.6) | 12(14.8) |  | 7(38.9) | 3(12.5) |  |

Note: Unless indicated otherwise, data are shown as number of patients, with the percentage in parentheses; #, Data are mean ± standard deviation; *, Data are medians, with interquartile ranges in parentheses. a, t-test; b, Mann-Whitney U-test; others (chi-square test or Fisher exact test). NOR, non-objective response; OR, objective response; HBsAg, hepatitis B surface antigen; BCLC, Barcelona Clinic Liver Cancer; AFP, alpha-fetoprotein; CEA, carcinoembryonic antigen; AST, aspartate transaminase; ALT, alanine transaminase; NLR, neutrophils/lymphocytes ratio; PLR, platelet/lymphocytes ratio.

**Table S2.** **Comparison between different CNN algorithm in TACE response prediction in multiple datasets.**

|  | Training set | Internal validation set | External test set 1 | | External test set 2 |
| --- | --- | --- | --- | --- | --- |
| **DenseNet169** |  |  |  |  | |
| AUC | **0.982** | **0.764** | **0.649** | **0.629** | |
| Accuracy | 0.943 | 0.705 | 0.652 | 0.595 | |
| Sensitivity | 0.959 | 0.775 | 0.745 | 0.618 | |
| Specificity | 0.940 | 0.756 | 0.566 | 0.639 | |
| **ResNet50** |  |  |  |  | |
| AUC | 0.950 | 0.756 | 0.652 | 0.591 | |
| Accuracy | 0.910 | 0.690 | 0.618 | 0.543 | |
| Sensitivity | 0.943 | 0.708 | 0.654 | 0.770 | |
| Specificity | 0.903 | 0.767 | 0.614 | 0.458 | |
| **ResNet18** |  |  |  |  | |
| AUC | 0.958 | 0.728 | 0.641 | 0.580 | |
| Accuracy | 0.909 | 0.624 | 0.606 | 0.535 | |
| Sensitivity | 0.938 | 0.716 | 0.678 | 0.697 | |
| Specificity | 0.912 | 0.744 | 0.600 | 0.527 | |
| **Transformer** |  |  |  |  | |
| AUC | 0.701 | 0.751 | 0.669 | 0.584 | |
| Accuracy | 0.671 | 0.600 | 0.622 | 0.609 | |
| Sensitivity | 0.601 | 0.692 | 0.670 | 0.558 | |
| Specificity | 0.773 | 0.744 | 0.645 | 0.649 | |

**Table S3** **Delong test for ROC improvements of DLTR_MLP_ compared to single-modality models in multiple cohorts.**

|  | Validation set | *P* value | External test set 1 | *P* value | External test set 2 | *P* value |
| --- | --- | --- | --- | --- | --- | --- |
| DLTR_MLP_ | / | / | / | / | / | / |
| DLTR_LR_ | 0.07 | 0.413 | 0.152 | 0.0015^a^ | 0.159 | 0.0724 |
| DLTR | 0.058 | 0.512 | 0.151 | 0.002^a^ | 0.189 | 0.0348^a^ |
| Clinical | 0.191 | 0.022 ^a^ | 0.213 | ＜0.001^a^ | 0.14 | 0.0625 |

Note: Data were metric value. ^a^ *P*＜0.05 indicated significant difference between models in the test.
